# An X chromosome‐wide DNA methylation study of Alzheimer’s disease

**DOI:** 10.1002/alz.091440

**Published:** 2025-01-09

**Authors:** Lily Wang, Wei Zhang, Lissette Gomez, Brian W. Kunkle, David Lukacsovich, Michael A. Schmidt, Achintya Varma, Anthony J. Griswold, William S. Bush, Eden R. Martin

**Affiliations:** ^1^ John P. Hussman Institute for Human Genomics, University of Miami Miller School of Medicine, Miami, FL USA; ^2^ University of Miami Miller School of Medicine, Miami, FL USA; ^3^ John P. Hussman Institute for Human Genomics, University of Miami Miller School of Medicine, Miami, FL, USA, Miami, FL USA; ^4^ Cleveland Institute for Computational Biology, Department of Population and Quantitative Health Sciences, Case Western Reserve University, Cleveland, OH USA

## Abstract

**Background:**

Sex is an important factor that contributes to both clinical and biological heterogeneity in Alzheimer’s disease (AD), but the regulatory mechanisms underlying sex differences in AD are still not well understood. DNA methylation (DNAm) is an epigenetic modification that regulates gene transcription and is known to be involved in AD. However, due to analytical and biological complexity, few previous DNAm studies analyzed the X chromosome, where many genes influencing cognitive abilities and immune functions are located.

**Method:**

We performed a sex‐specific X chromosome‐wide analysis of the DNAm data generated by the longitudinal Alzheimer’s Disease Neuroimaging Initiative (ADNI) study. We used mixed effects logistic regression models with AD status as the outcome, adjusted for age, sex, batch, and immune cell‐type proportions, random subject effects, and corrected for inflation.

**Result:**

Our analysis included 632 female DNAm samples (188 cases, 444 controls) and 652 male DNAm samples (239 cases, 413 controls), measured on blood samples of 179 and 219 independent subjects with ages older than 65 years. Given our modest sample size, we considered CpGs with suggestive significance at the prespecified significance threshold of P < 1×10^‐5^. In females, we identified 2 significant CpGs (cg04150893 and cg16580361), mapped to the intergenic region and gene body of the HMGN5 gene. No significant CpGs were identified in male samples. Interestingly, blood DNAm at cg16580361 is significantly associated with DNAm in the prefrontal cortex (Blood Brain DNA methylation Comparison Tool: r = 0.599, P = 1.74×10^‐8^). Consistent with our observed hypermethylation at cg16580361 in ADNI data (OR = 1.11, P = 6.14×10^‐6^), the HMGN5 gene is also significantly upregulated in the frontal cortex of female AD subjects (Agora database https://agora.adknowledgeportal.org: OR = 2^0.227^ = 1.17, adjusted P = 5.7×10^‐6^). The HMGN5 gene is involved in the metabolism of the brain antioxidant glutathione. Decreased levels of glutathione have been implicated in both AD onset and progression.

**Conclusion:**

Our analysis of the X chromosome in the ADNI study dataset nominated cg16580361 located on the HMGN5 as a plausible biomarker for AD. Future studies that validate our findings in larger and more diverse community‐based cohorts are needed.

